# Immune-driven stromal inflammation in pancreatic cancer within a microfluidic platform

**DOI:** 10.1088/1758-5090/ae7b09

**Published:** 2026-06-26

**Authors:** Mariana Viso, Karthik Rajkumar, Anna Bianchi, Bhumi Suthar, Neil Kumar, David Oliver, Mya Collins, Jashodeep Datta, Ashutosh Agarwal

**Affiliations:** 1 Department of Biomedical Engineering, University of Miami, Coral Gables, FL, United States of America; 2 Division of Surgical Oncology, Dewitt Daughtry Department of Surgery, University of Miami Miller School of Medicine, Miami, FL, United States of America; 3 Department of Neuroscience, Brown University, Providence, RI, United States of America; 4 Sylvester Comprehensive Cancer Center, University of Miami Miller School of Medicine, Miami, FL, United States of America; 5 Desai Sethi Urology Institute, University of Miami Miller School of Medicine, Miami, FL, United States of America

**Keywords:** cancer spheroid, cancer associated fibroblast, pancreatic cancer, microfluidics, tumor microenvironment, organs on chips

## Abstract

Pancreatic ductal adenocarcinoma (PDAC) is defined by a highly fibrotic and immunosuppressive tumor microenvironment (TME). The inflammatory polarization of cancer-associated fibroblasts (CAFs), as well as the dense and early trafficking of myeloid-derived suppressor cells (MDSCs) contribute to tumor progression, immune exclusion, and therapy resistance. Yet, key mechanisms governing their interplay remain undefined. We developed a novel microfluidic platform that recapitulates the immuno–stromal PDAC microenvironment and enables controlled co-culture, high-resolution live imaging, and downstream molecular analyses. Spheroids composed of tumor cells, and dual reporter CAFs, were embedded in our engineered system and allowed to dynamically interact with MDSCs for 48 h. Dynamic interactions with MDSCs induce a significant increase in inflammatory CAF-specific reporter fluorescence compared to media controls. Gene set enrichment analysis demonstrated enrichment of inflammatory response pathways, and increased expression of iCAF related genes. RNA velocity, CellRank, and PAGA trajectory analyses collectively identify a constitutively fate-committed CAF progenitor population that is selectively expanded by MDSC exposure and preferentially transitions into an inflammatory iCAF state. Overall, our novel microfluidic platform recapitulated targeted MDSC-driven inflammatory CAF polarization and revealed a progenitor CAFs population as a potential precursor for iCAF differentiation, establishing its utility in mechanistic studies of stromal–immune interactions of the TME.

## 1. Introduction

Eighty-seven percent of patients diagnosed with pancreatic ductal adenocarcinoma (PDAC) die within the first 5 years after diagnosis [1–3], making it one of the deadliest cancers. The poor prognosis arises in part due to the limitations of current treatments [4, 5]. While chemotherapy and surgical resection are the current standard of care for PDAC patients who present with localized disease, their effectiveness is severely hampered by the rapid development of chemoresistance [6, 7]. The PDAC tumor microenvironment (TME) is a complex, inflammatory and immunosuppressive ecosystem that houses the pancreatic tumor, allowing it to grow and metastasize while hindering the effectiveness of chemo and immunotherapies [8–10].

A key cellular constituent in the PDAC TME that drives therapeutic resistance is the cancer-associated fibroblast (CAF) compartment. Inflammatory CAFs (iCAFs) are central drivers of pro-inflammatory signaling and immunosuppression [11, 12]. Through abundant cytokine and chemokine secretion, including IL-6 and LIF, iCAFs are able to beckon suppressive innate immune cells such as myeloid-derived suppressor cells (MDSCs) to the TME [13–15]. iCAFs thus maintain a chronic inflammatory milieu that ultimately promotes cancer cell progression by enhancing proliferation and epithelial-to-mesenchymal transition [8, 16]. The relationship between iCAFs and MDSCs has largely been characterized in one direction, where iCAF-derived signals govern MDSC recruitment and retention within the TME [12, 17, 18]. However, whether this relationship is truly bidirectional, and specifically whether MDSCs possess the capacity to act upon resident fibroblasts and drive their polarization toward a pro-inflammatory iCAF state, remains unanswered. Emerging evidence suggests that MDSCs and iCAFs engage in reciprocal crosstalk, yet the key mechanisms by which MDSCs specifically drive iCAF induction remain undefined [19–22]. This highlights a critical gap that limits our ability to identify new therapeutic targets for patients with PDAC.

Current models present significant challenges for studying the dynamic immune–stromal crosstalk that governs PDAC development and therapeutic resistance. *In vivo* systems like patient-derived xenografts lack a comprehensive immune compartment, limiting their applications for observing the immunological relationships that impact PDAC [23]. Other models, such as genetically engineered mouse models [24, 25], capture many aspects of PDAC pathology, including the immunological response to the tumor, but are limited by complex overlapping signaling pathways that hinder elucidation of the isolated contributions of heterocellular crosstalk (e.g. MDSC–CAF) to tumor progression in real-time [26, 27]. In contrast, *in vitro* systems such as two-dimensional monolayers provide control and accessibility but fail to capture the three-dimensional architecture and dynamism of TME crosstalk in PDAC. Without this spatial complexity, they cannot reproduce critical features such as oxygen gradients, or the compartmentalized infiltration of immune cells that drive tumor progression [28, 29]. Even three-dimensional cultures, while more physiologically relevant, often lack the perfusion needed to prevent waste build up and necrosis [30, 31]. These *in vitro* systems also neglect to include the dynamic component needed to mimic immune cell migration and infiltration found *in-vivo*.

This critical gap in our understanding of PDAC immune–stromal dynamics highlights the need for systems that will facilitate the study of these mechanisms to uncover potential therapeutic targets to attenuate tumor growth and improve patient survival. Microphysiological systems (MPS) can mitigate these pitfalls by enabling real-time demonstration of specific PDAC cellular interactions while maintaining the three-dimensional architecture and dynamic cues of the *in vivo* TME. While microfluidic platforms have previously been utilized to study the tumor–immune interactions in a wide range of cancers [32–42], few have captured the dynamic interplay between the TME-derived inflammatory and immunosuppressive networks that define PDAC.

In this study, we developed a novel microfluidic platform that recapitulates multiple *in vivo* features of PDAC, including the three-dimensional tumor–stromal architecture, dynamic perfusion and immune cell infiltration, and real-time MDSC–CAF interactions while maintaining precise control over cellular composition that will allow investigation of the critical mechanisms of TME-derived crosstalk driving tumor progression and therapeutic resistance.

Unlike most MPS, which are permanently sealed, this novel platform allows for the extraction of the cellular components for terminal assays. Additionally, it allows for real-time observation of the immune cells as they flow through the system and interact with the tumor-CAF clusters. We demonstrated that MDSCs play a key role in pushing CAF polarization towards an inflammatory state, identified a metabolically primed precursor CAF population that may serve as a transitional state for iCAFs, and found evidence that MDSCs exert targeted interactions with this subset to shape stromal composition. By identifying a metabolically primed precursor CAF population and its targeted interaction with MDSCs, this study provides a new cellular target for future therapeutic intervention, opening avenues for the design of therapeutic agents that may interrupt immuno–stromal crosstalk and improve PDAC patient survival.

## 2. Materials and methods

### 2.1. Computational modeling of fluid dynamics

STEP file of the three-dimensional model of the channels in the platform, along with an approximate spheroid embedded on the center of the well, was exported to COMSOL Multiphysics (COMSOL Inc. Sweden) for finite element analysis. Fluid flow through the channels was modeled as water and governed by the Navier–Stokes equation, with a flow rate of 10 *µ*l min^−1^ to match our *in vitro* parameters. Fluid dynamics within the platform were modeled using laminar flow and transport of diluted species physics modules. Velocity and shear stress values across the channel height were exported to compare with the *in-*vitro particle imaging velocimetry (PIV) values and graphed using BioRender Data Analysis and visualization (BioRender, Canada).

### 2.2. Microfluidic platform design and fabrication

**Figure 1.**
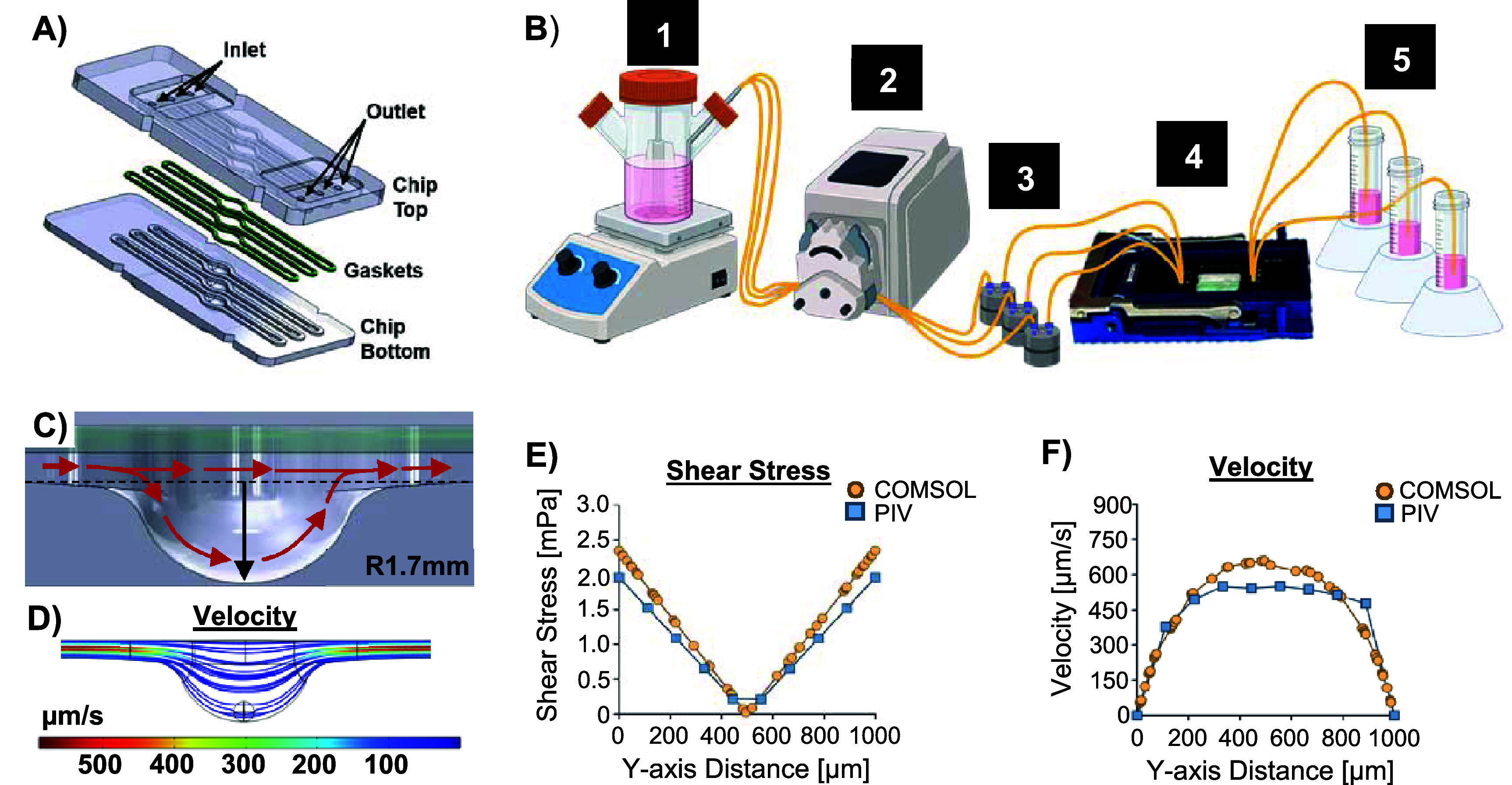
System design and set-up. (A) Exploded view of SolidWorks design, including the top and bottom pieces, as well as the gaskets. (B) Diagram of running parameters. The flow from a spinner flask (1) is controlled by a peristaltic pump (2) and passed by debubblers (3), before reaching the chip (4). The outflow of the chip is collected in glass vials (5) for further testing. (C) Cross-section of concaved multi-well plates commercially used to create structurally stable spheroids. Red arrows are used to mimic flow. (D) COMSOL analysis of the velocity fields across the platform’s concaved channels (m s^−1^) with a spheroid, demonstrating full well perfusion. Graph showcasing the shear stress (E) and velocity (F) across the channel’s height, compared to COMSOL simulations.

Both the top and the bottom sections of our microfluidic platform, as well as our gasket molds, were designed in SolidWorks. Each design was then individually micro-milled into UV/scratch resistant polymethyl methacrylate (PMMA) sheets (McMaster, C# 8560K257, 12”x24”x1/8”) using a MDX-540 CNC milling machine (Roland, Japan). Lastly, the platform was cut to fit chip clamps (Micronit Microfluidics, Netherlands) using a 30 W CO_2_ laser engraver (Epilog Laser, United States). Duraseal 1533 silicone epoxy (Cotronics Corp., United States) was used to create the silicone gaskets used to seal our platform (figure 1(A)).

### 2.3. PIV

Tracer solution was made with 4 *µ*m diameter FluoSpheres (Thermo F8859) at a concentration of 5 000 000 particles ml^−1^ in distilled water. Velocity was maintained at 10 *µ*l min^−1^ and run for 3 h. The platform was run at room temperature using the synchronized video microscope 340 (Lab Smith, United States) with a 4x magnification. The channel was divided into eight equal-height boxes, stacked vertically during analysis. Average velocity across these boxes was used to analyze the fluidic path at different heights across the channel. Average velocity and shear stress calculated from the software across all eight points were graphed using BioRender data analysis and visualization (BioRender, Canada). To correct for systematical discrepancies of PIV measurements, such as limited spatial resolution, and experimental noise, compared to COMSOL simulations, PIV-derived data was scaled by a factor of three to match the simulation values.

### 2.4. Cell lines and cell culture

All cell lines were cultured at 37 °C and 5% CO_2_ in DMEM media with GlutaMAX, 10% fetal bovine serum and 1% penicillin–streptomycin. Cells were frozen between passage 3–7, and no cell lines were used after 15 passages. J774M cells were used as MDSCs for these experiments, as they have been shown to recapitulate the phenotype and function of polymorphonuclear MDSCs (PMN-MDSCs) *in vitro* [43]. J774M cells, a murine PMN-MDSC-like cell line derived from the J774 myeloid cell line by sorting of CD11b^+^Gr-1^+^ cells, were kindly provided by Dr Jaffe, John Hopkins University. Prior to use in experiments, J774M cells were confirmed to express canonical PMN-MDSC surface markers Ly6G and Gr-1 by flow cytometry, consistent with the established phenotypic definition of murine PMN-MDSCs [44, 45]. Dual-reporter CAF (DR-CAFs) were kindly gifted by Dr Schworer, University of Chicago. DR-CAFs were engineered, using pancreatic stellate cells (PSCs), to monitor both myofibroblastic and inflammatory CAF activation states. PSCs were isolated from *α*SMA-DsRed mice, leading to red fluorescence following Acta2 promoter expression [46]. Isolated cells were then transduced with a lentiviral reporter encoding enhanced green fluorescent protein (EGFP) under the murine IL6 promoter (pmIL6promoter-EGFP)[46]. The K8484 cell line was derived from pancreatic tumors arising in KPC (LSL-Kras^G12D/+^; LSL-Trp53^R172H/+^; Pdx1-Cre) mice via collagenase digestion and serial passage, as previously described [47]. Cells were obtained as a kind gift from David Tuveson (Cold Spring Harbor Laboratories) and maintained in DMEM supplemented with 10% FBS. This cell line was used to model PDAC tumor cells and is referred to in this manuscript as KPC tumor cells.

### 2.5. Spheroid formation

Twenty-four hours prior to seeding the chips, cultured DR-CAFs and KPC tumor cells are passaged and counted. A cell suspension was prepared such that each 200 *µ*l of solution contains 6000 DR-CAFs and 2000 KPC tumor cells. A 3:1 CAF to tumor cell ratio was selected to reflect the well-established stromal dominance of the PDAC microenvironment. In PDAC, stromal components (including cellular and noncellular components) can comprise up to 80% of the total tumor volume [48]. While no standardized tumor-to-CAF ratio has been established due to the heterogeneity of patient tumors, this ratio was chosen to reflect the dominant contribution of CAFs while maintaining spheroid size suitable for imaging and downstream analyses. 200 *µ*l of the cell solution is added into each well of an ultra-low attachment 96-well plates (C# 3830 Corning, United States). The ultra-low attachment surface is coated with a hydrophilic, non-ionic hydrogel that prevents cell adhesion to the plate substrate, thereby eliminating cell-surface interactions and promoting spontaneous self-aggregation of cells into compact three-dimensional spheroids without the requirement for Matrigel, scaffold embedding, or mechanical agitation. Plates were incubated overnight at 37 °C and 5% CO2 to allow for spontaneous spheroid formation. Spheroid compaction and morphology were confirmed by brightfield microscopy prior to use in downstream experiments.

### 2.6. Chip operation

Running parameters shown in figure 1(B) were first assembled inside biosafety cabinets, and then transferred to incubators set to 37 °C and 5% CO2, for a 48 h run. We selected 48 h because it provided the clearest separation between conditions (largest dynamic range in iCAF/myCAF ratio), while avoiding the low signal at 24 h and the reduced divergence of treatment and control samples at 72 h (supplementary figure 1). First, 100 *µ*l of a running cell solution with a concentration 200 000 MDSCs per ml of media was prepared in our 125 ml spinner flask (Corning, United States) and transferred to the incubator. At higher MDSC dosages (ex. 1000 000 cells ml^−1^) we observed rapid disintegration of spheroids and limited myCAF-associated fluorescence (supplementary figure 2). Next, a pump tubing (Masterflex, Germany) was connected to a 0.25 mm inner diameter FEP fluoropolymer tubing, which was then passed through our Reglo independent channel control peristaltic pump (Masterflex, Germany) and connected in the following order: Diba Omnifit High Flow Bubble Trap (Cole-Parmer, England), Micronit clamp (Micronit Microfluidics, Netherlands), and outlet reservoir. This was done 3 times to create a fluidic pathway for each of the platform’s channels. Once this fluidic pathway was assembled, it was set on the incubator, and the end of the pump tubing was introduced into the spinner flask. Gaskets were then placed on the top piece of the platform, and spheroids were gently moved to each well of the bottom piece of the platform. The pieces were then closed and sealed with the Micronit clamp (figure 1(B)). Same steps were followed for the single-cell RNA sequencing samples, except that the running cell solution concentration was decreased to 20 000 MDSCs per ml and five spheroids were introduced per well. This was done in order to ensure we primarily capture PDAC tumor cells and CAFs for our analysis.

Inflammatory controls for fluorescence experiments were made using standard DMEM media with TNF*α* concentration of 800 *µ*g ml^−1^ (R&D Systems, United States), as anything above this level led to issues with spheroid integrity over time. Non-inflammatory controls for fluorescence experiments were made using standard DMEM media with TGF*β* concentration of 125 *µ*g ml^−1^ (R&D Systems, United States), as anything above this level did not significantly change the level of myCAF polarization.

### 2.7. Fluorescence imaging and analyses

Fluorescence imaging was performed using a LSM 700 confocal laser scanning microscope (Zeiss, Germany) to quantify polarization of inflammatory CAFs in our spheroids. Spheroids were transferred to a 96-well optical-bottom microplate (Nunc Solutions, Denmark) and imaged under identical settings for laser power, gain, and exposure to ensure comparability across conditions. Z-stack images were acquired and maximum intensity projections generated for analysis.

Grayscale images for each sample were created using ImageJ. We developed a MATLAB script to quantify the number of pixels above a defined fluorescence threshold in each image. The threshold was established by identifying pixels in the grayscale images that were visually determined by the user to represent true fluorescence. Samples for all four groups were analyzed together, and group’s information was removed during threshold selection for each image, to minimize bias.

### 2.8. Single cell RNA sequencing and analysis

After the 48 h run, all 15 spheroids for each group were removed from the platform and transferred to a 1.5 ml tube using PBS. Spheroids were centrifuged at 420xg for 4 min and resuspended in 500 *µ*l of 0.25% trypsin. They were then incubated for 3 min at 37 °C and 5% CO2. Clumps were removed by gently pipetting up and down the solution and adding 500 *µ*l of media. Samples were once again centrifuged at 420xg for 4 min and resuspended in 500 *µ*l of BSA buffer. Finally, samples were filtered using a filter tube and centrifuged at 200xg for 4 min to remove any debris or dead cells, before being resuspended in 100 *µ*l of BSA buffer.

It should be noted that each experimental condition was represented by a single pooled sequencing library generated from 15 spheroids, as individual spheroids did not yield sufficient cell numbers to meet the minimum input requirements of the 10× Genomics platform. Single-cell suspensions were assessed for viability and processed for single-cell RNA sequencing library construction by the Oncogenomics-Shared Resource Facility (University of Miami). Sequencing libraries were generated using the Chromium 85 Controller, Chromium Next GEM Single Cell 3’ GEM, Library & Gel Bead Kit v3.1, and Chromium Next GEM Chip G kit (10× Genomics). The cDNA and libraries were sequenced on an Illumina NovaSeq 6000 to achieve sufficient read depth per cell. Raw sequencing data were processed using Cell Ranger (version 7.0, 10× Genomics) for alignment, UMI counting, and gene expression quantification.

Downstream analyses, including normalization, clustering, and marker identification, was performed using Seurat (v4.3) in R (v4.4.2). Cells expressing less than 200 or more than 2500 genes, as well as those that had more than 10% mitochondrial gene expression were filtered out from analysis. Cells were normalized to the total cells isolated for each group. Cells with high mitochondrial content or low gene counts were excluded, and visualizations such as uniform manifold approximation and projection (UMAP) or feature plots were generated to examine cell-type-specific expression patterns. Data normalization was performed using the LogNormalize method with a scale factor of 10 000. The top 2000 variable features were identified using the VST selection method. Principal components were retained based on a 90% variance threshold, confirmed by elbow plot inspection, resulting in approximately 10 principal components. Clustering was performed at a resolution of 0.3 using the FindClusters function. UMAP visualization was generated using the retained principal components with default Seurat parameters (n.neighbors = 30, min.dist = 0.3). Gene set enrichment analysis (GSEA) was performed using the fgsea (R studio, v1.32.4) clusterProfiler (R studio, v4.14.6) and enrichment (R studio, v1.26.6) packages. A ranked gene list, ordered by log_2_ fold-change from the edgeR results, was used as input for enrichment testing. We evaluated pathway enrichment using the MSigDB Hallmark gene sets, restricted to the mouse ortholog versions provided through the msigdbr database. Normalized enrichment scores, nominal *p* values, and FDR-adjusted *q* values were reported, and gene sets with FDR < 0.25 were considered significantly enriched.

Spliced and unspliced RNA counts were extracted from Cell Ranger BAM files using Velocyto (v0.17) with the mm10 annotation and integrated with Seurat cluster labels for analysis in scVelo (v0.3.x). Genes were filtered to a minimum of 20 shared counts, normalized, and the top 2000 highly variable genes were selected. RNA velocity was estimated using the dynamical model with moments computed across 30 principal components and 30 nearest neighbors. PAGA transition probabilities were computed using the dynamical velocity kernel; consistent with Wolf *et al*, who introduced PAGA and recommended discarding low-weight edges to reveal denoised network topology, transitions with scores and differences in scores below 0.15 were considered below the threshold for meaningful connectivity [49]. Cell fate probabilities were estimated using CellRank’s Markov chain framework (n_states = 4), identifying clusters 4 and 2 as terminal states. Pseudotime ordering between clusters 6 and 4 was assessed using a two-sided Mann–Whitney *U* test applied to latent time distributions per condition.

## 3. Results

### 3.1. Platform maintains through perfusion and laminar flow throughout the channel

We sought to create a platform that supports three-dimensional structures of tumor-CAF spheroids, while maintaining full perfusion and laminar flow of MDSCs throughout the system. Conventional microfluidic device fabrication methods rely on planar lithography and polydimethylsiloxane (PDMS) casting, which constrain the system’s architecture to planar geometries, thus making it impossible to create concave structures. Using precision micro milling of bioinert acrylic sheets, we created concaved wells for each channel (figure 1(C)) to recreate current commercial standards for maintaining spheroid integrity and preventing disassociation [50]. Additionally, this manufacturing technique allowed us to create a resealable two-piece platform from which we can extract samples for downstream terminal assays. Finite element analysis was used to demonstrate that the addition of this new geometry was capable of maintain full perfusion throughout the whole well (figure 1(D)). Additionally, we observed that the system still maintained a low enough velocity that the spheroids will not be pushed out or subjected to significant shear stress (figure 1(D)). PIV was then used to validate that a stable laminar flow was maintained throughout the channel shown in figure 1(F) and reinforced the results of COMSOL simulation. Shear stress measurements followed laminar flow characteristics, as it maintained low stress towards the midline (<0.3 mPa) and elevated stress near the boundaries (∼2.0 mPa) (figure 1(E)). Velocity maximums were achieved at the midline (∼550 *µ*m s^−1^), with velocities decreasing closer to the walls of the system, consistent with the laminar distribution predicted by COMSOL (figure 1(F)). PIV results (figures 1(E) and (F)), confirming that laminar flow was maintained throughout the channel geometry. These results validated both our computational models and our platform design, demonstrating our platform’s capabilities to reliably maintain perfusion for nutrient-waste exchange, without exposing seeded spheroids to damaging shear stress or significant motion artifacts.

### 3.2. MDSCs induce an increase in inflammatory CAF-specific fluorescence levels

**Figure 2.**
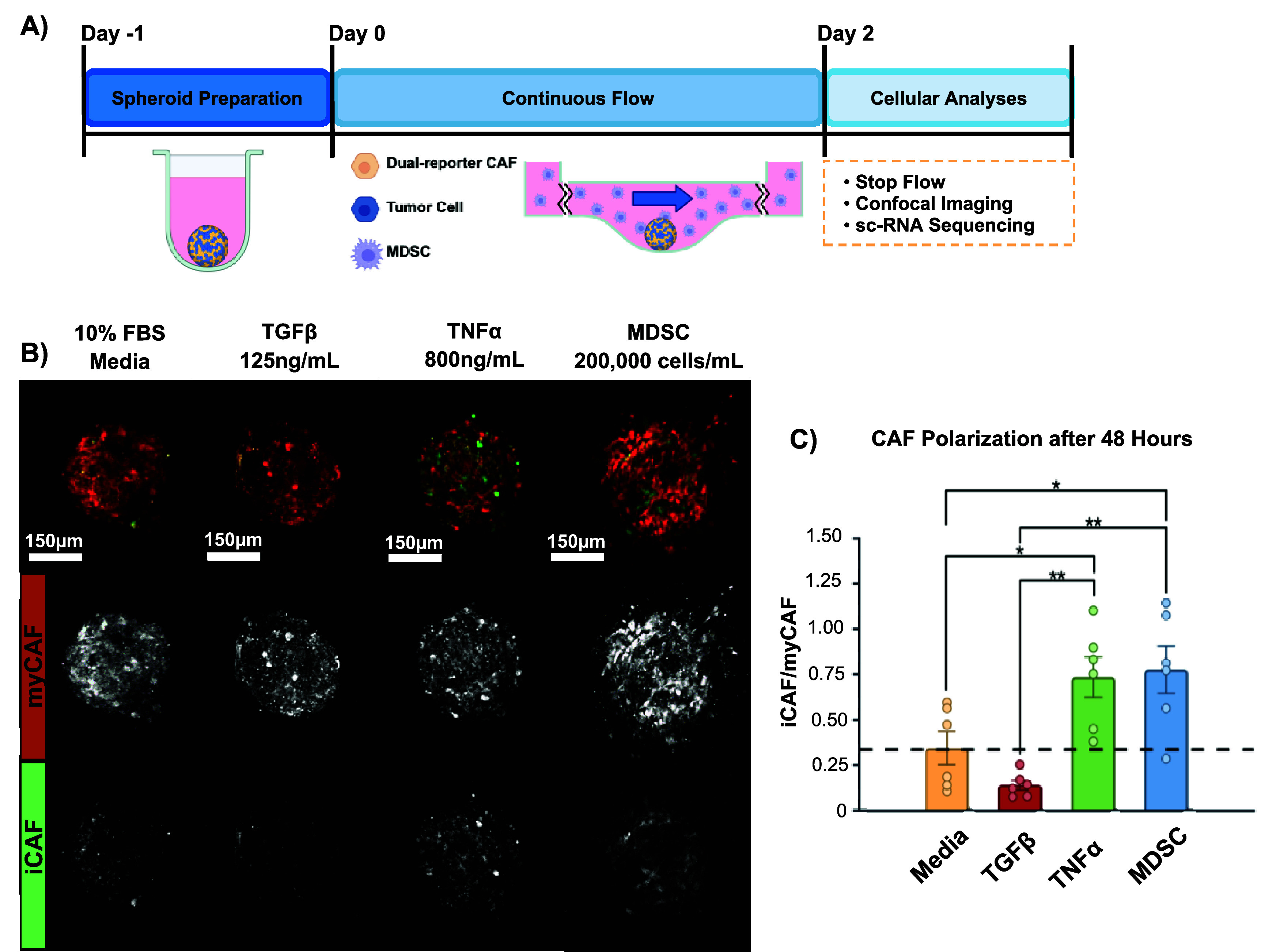
Fluorescence based characterization of CAF polarization. (A) Spheroids were prepared 24 h before they were to be introduced into the chip. Once in the chip, flow was maintained for 48 h at 10 *µ*l min^−1^. Afterwards, spheroids were extracted and analyzed. (B) Confocal images of spheroids following 48 h of incubation under different conditions. Red fluorescence indicates myCAF configuration and green indicates iCAF configuration. Merged channels (top), isolated 594 fluorescence (middle), and isolated 488 fluorescence (bottom). (C) Graph showing the ratio of iCAF to myCAF fluorescence. No significant difference was observed between media and TGF*β*, as well as between TNF*α* and MDSCs. Data shown are mean $ \pm $ SEM. Statistical significance was done using Welch’s *t*-test. ** represents *p* ⩽ 0.01, * represents *p* ⩽ 0.05. Each group had an *n* = 6, representing individual spheroid measurements.

We investigated if MDSCs could modulate CAF polarization by imaging and quantifying the intensity of fluorescence indicative of either inflammatory (iCAF; green) or myofibroblastic (myCAF; red) differentiation, following 48 h of incubation under different treatment conditions (figure 2(A)). Spheroids perfused solely with 10% FBS media served as our experimental control, which exhibited minimal inflammatory polarization (figure 2(B)), with a low iCAF/myCAF ratio (figure 2(C)). To generate a non-inflammatory control for our study, we perfused spheroids with TGF*β*, as this cytokine is known to promote the polarization of fibroblasts into myCAFs [51–53]. Spheroids perfused with media and TGF*β* exhibit minimal inflammatory polarization, with low iCAF/myCAF ratio (figure 2(C)). Media and TGF*β* control’s ratios showed no significant difference between them, demonstrate our platform’s ability to maintain stable perfusion of our spheroids without introducing non-specific inflammatory effects.

To create a pro-inflammatory control, we perfused spheroids with TNF*α*, an established driver of iCAF differentiation [53–57]. This group of spheroids showed strong inflammatory fluorescence (figure 2(B)), with a statistically significant 2.6-fold increase (*p* < 0.01) in iCAF/myCAF ratio compared to the media control (figure 2(C)). Notably, spheroids perfused with MDSCs displayed similarly high levels of inflammatory CAF polarization (figure 2(B)), with an iCAF/myCAF ratio statistically similar to TNF*α* control (figure 2(C)). These findings not only demonstrate the role of MDSCs in driving CAFs toward an inflammatory state, but the platform’s ability to replicate physiologically relevant dynamic cell-to-cell interactions as well. Importantly, by maintaining spheroid architecture, perfusable gradients, and controlled spatial organization of CAFs, tumor cells, and MDSCs, our platform allows for dynamic signaling and direct cell–cell interactions that closely mimic the *in vivo* TME. These features are largely absent in traditional 2D cultures where cells grow on flat, unstructured surfaces and lack physiologically relevant signaling gradients.

### 3.3. MDSCs induce CAF inflammatory polarization

**Figure 3.**
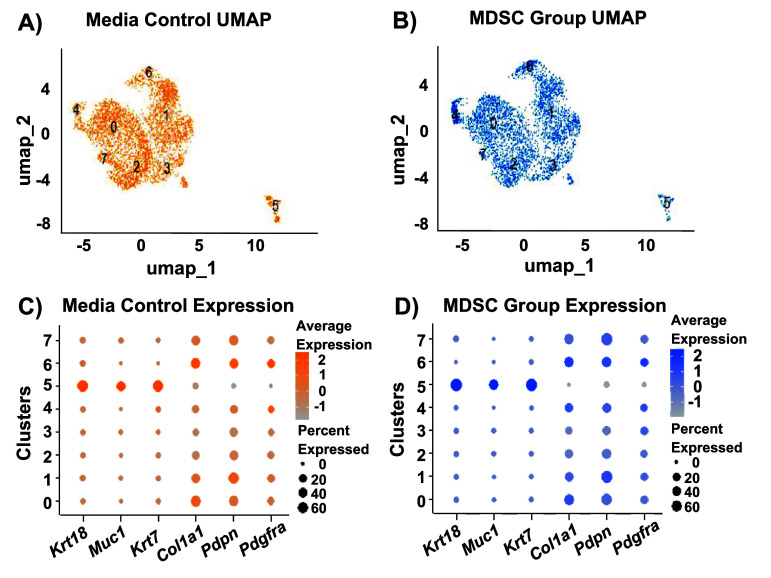
scRNAseq analyses. UMAPs of media-treated control spheroids for our media control group (A) and our MDSC-treated group (B). (C) Bubble plot for the expression level of media-treated samples for canonical marker genes of CAFs (Pdpn, Col1a1, and Pdgfra) and tumor cells (Krt18, Muc1, and Krt7). F) Bubble plot for the expression level of MDSC-treated samples for canonical marker genes of CAFs (Pdpn, Col1a1, and Pdgfra) and tumor cells (Krt18, Muc1, and Krt7).

We sought to characterize the dynamic changes in transcriptional heterogeneity in CAFs and tumor cells following interactions with MDSCs. To accomplish this, we perfused spheroids with either media or MDSC-treated conditions over 48 h on the platform (figure 2(A)) and retrieved the spheroids for single cell RNA sequencing (scRNAseq). In order to capture tumor-CAF changes invoked by MDSCs, we reduced MDSC flow concentration to 20 000 cells ml^−1^ to ensure primarily the capture of PDAC tumor cells and CAFs. Clustering and visualization using UMAP 7 cell clusters for CAFs (1–4, and 6–7), as well as a single cell cluster (cluster 5) of tumor cells (figures 3(A) and (B)). CAF clusters were identified using known markers *Pdpn, Col1a1* and *Pdgfra* [58–62], and tumor cell clusters were marked by the expression of *Krt18, Muc1*, and *Krt7* (figures 3(C) and (D))[63–65].

We then compared the cell distribution across each cluster, to uncover the presence of enriched clusters in both our media control and MDSC group and found that MDSC treatment significantly altered the distribution of cells across clusters compared to media control (chi-square test, *χ*^2^(7) = 335.9, *p* < 0.0001). The most striking changes were the 8.65-fold expansion of cluster 4 and the 4.69-fold expansion of cluster 6 for the MDSC group, compared to the media control. All remaining CAF clusters showed proportional changes of less than 1.25-fold in either direction. To further investigate the gene signature of cluster enriched in the control and MDSC groups, we performed GSEA.

### 3.4. MDSCs promote progression of CAF progenitors toward an inflammatory landscape

**Figure 4.**
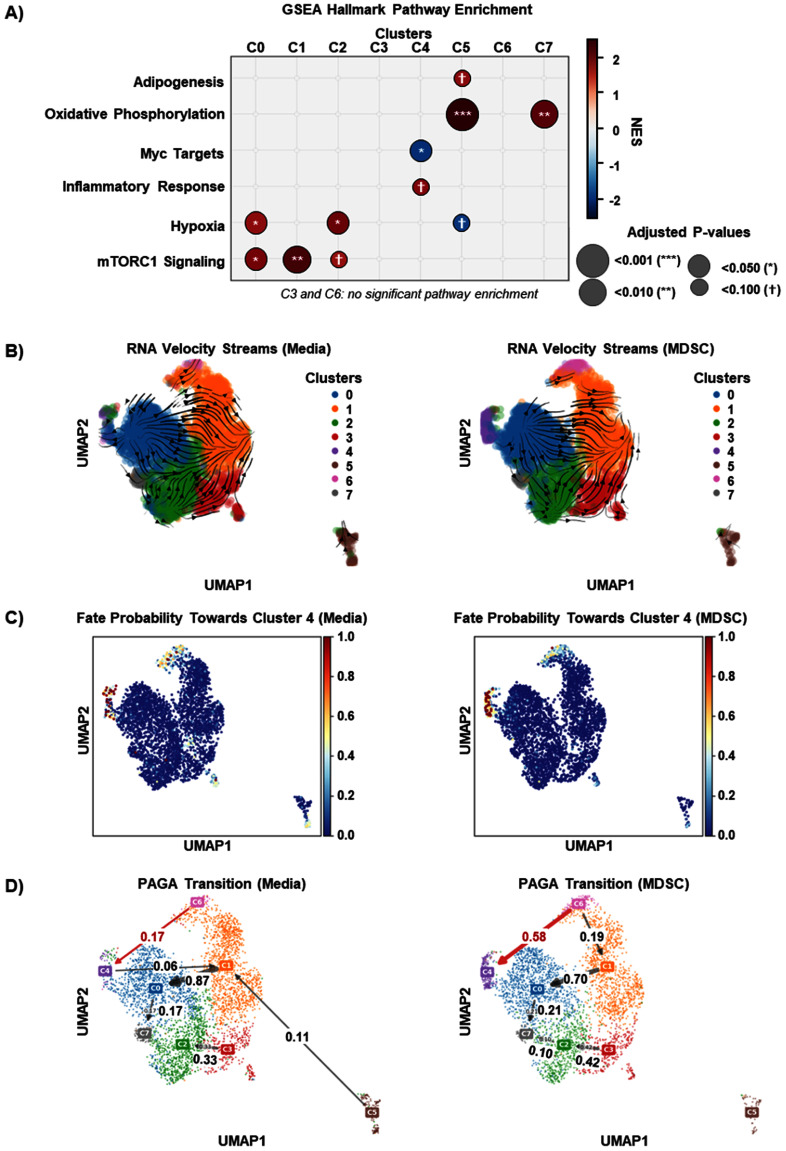
RNA Velocity & Trajectory. (A) Bubble plot of GSEA pathways enriched across clusters, including the adjusted p-values and normalized enrichment scores (NES) for each pathway. For all the following panels, media control graph is shown on the left, and the MDSC group is shown on the right. (B) UMAP with velocity stream arrows overlaid, colored by cluster. (C) UMAP colored by fate probability toward the cluster 4. (D) PAGA score graph showing edge weights.

GSEA analysis showcased a coordinated metabolic reprogramming across the CAF compartment, following MDSC exposure. Clusters 0 and 1 each activated mTORC1 signaling, and cluster 2 showed trending upregulation of mTORC1 signaling. Both clusters 0 and 2 also upregulated hypoxia pathways (C0 NES = 1.79; C2 NES = 2.07), as shown in figure 4(A). Together with the upregulation of glycolytic effectors (Hk2, Slc2a1, Gpi1) and stress-adaptive mediators (Hspa5, Ero1a, Hmox1) in clusters 0 and 2, these results suggests a coordinated anabolic reprogramming event in which MDSCs may be conditioning these CAFs toward a metabolically activated, stress-resilient state. In contrast, GSEA of cluster 4 CAFs in MDSC-treated spheroids showed trending upregulation of inflammatory response pathways (NES = 1.92) and downregulation of Myc target genes (NES = − 1.89), as shown in figure 4(A), compared to the media control. These transcriptional changes suggest a shift away from proliferative and growth programs toward an inflammatory state, consistent with the trending enrichment of inflammatory response pathways of cluster 4. Cluster 5, our only tumor cluster, upregulated oxidative phosphorylation (NES = 2.48) and trended toward downregulating hypoxia signaling (NES = − 1.80) when co-incubated with MDSCs (figure 4(A)), suggesting a metabolic shift toward mitochondrial oxidative phosphorylation in tumor cells under MDSC exposure. Cluster 6, despite its near 5-fold expansion, lacked significant pathway enrichment, thus raising the question of whether this population represents a transitional progenitor state, rather than a functionally independent subpopulation.

Given the dramatic expansion of both clusters 4 and 6 under MDSC treatment, we next performed RNA velocity analysis using the scVelo dynamical model to determine whether these two populations represent sequential states rather than independent subpopulations (figure 4(B)). Velocity confidence scores, were highest for cluster 6 in both the media control (0.893) and MDSC-treated (0.896) conditions, indicating that cluster 6 cells share a highly coherent transcriptional direction, moving consistently toward a common fate. CellRank Markov chain fate probability analysis (figure 4(C)) further reinforced cluster 6’s transcriptional momentum results. Cluster 6 cells carried a 90.3% probability of adopting the cluster 4 terminal state, nearly matching cluster 4’s self-fate probability of 91.7%, and far exceeding probabilities for other clusters (range 0.537–0.643). Notably, cluster 6 fate probability toward cluster 4 was similarly high under media control conditions (90.5%), as shown in figure 4(C), indicating that cluster 6 cells are constitutively committed to the inflammatory trajectory of cluster 4 and that MDSC co-incubation expands this population rather than redirecting it. Additionally, MDSC exposure modestly but consistently increased fate probability toward cluster 4 across all remaining CAF clusters (C0: 0.433→0.567; C1: 0.393→0.607; C2: 0.463→0.537; C3: 0.402→0.598; C7: 0.436→0.564).

To further investigate the structural connectivity between clusters, and strengthen CellRank results, we performed PAGA transition analysis (figure 4(D)). PAGA transition analysis revealed a dominant cluster 1 to cluster 0 transition in both conditions, reflecting the strong transcriptional connectivity between these two clusters that remained the strongest connection in the network under both conditions. While MDSCs drive metabolic reprogramming in cluster 0 and cluster 1 through mTORC1 and hypoxia pathway activation, neither their abundance nor their dominant connectivity with each other was altered, suggesting these populations are transcriptionally activated in place rather than transitioning to a new state. Most strikingly, the cluster 6 to cluster 4 transition probability increased 3.4-fold under MDSC treatment, representing the largest condition-dependent change in the entire transition network and directly reflecting the accelerated flux of progenitor cells toward the cluster 4 state. Additionally, a new, yet modest, cluster 6 to cluster 1 connection emerged exclusively under MDSC treatment, suggesting that MDSC exposure may broaden the transitional output of cluster 6 beyond the primary C4 trajectory, potentially diverting a subset of activated progenitor cells toward an alternative early-cluster fate. To determine the developmental ordering underlying the massive cluster 6 to cluster 4 transition identified by PAGA, we next performed pseudotime and latent time analysis using the scVelo dynamical model. Both analysis placed both cluster 6 and cluster 4 at late positions within the trajectory (supplementary figure 3), with the Mann–Whitney *U* testing confirmed that cluster 6 cells are positioned significantly earlier than cluster 4 cells under MDSC treatment (*p* = 0.000022, two-sided), with a trend toward earlier positioning also observed under media control conditions (*p* = 0.070, two-sided).

Taken together, these findings support a model in which MDSCs drive a coordinated remodeling of the CAF compartment through two complementary mechanisms. First, MDSCs broadly activate the transcriptional state of existing CAF populations, inducing mTORC1 and hypoxia signaling across clusters 0, 1, and 2, while simultaneously shifting cluster 4 away from proliferative programs, by suppression of MYC targets and trending enrichment of inflammatory response pathways. Second, MDSCs selectively expand a transcriptionally committed cluster 6 progenitor population, amplifying a pre-existing differentiation trajectory toward cluster 4. Additionally, compartment-wide increase in fate probability toward cluster 4 across all remaining CAF clusters further suggests that this remodeling extends beyond the cluster 6, pointing to a compartment-wide shift toward the cluster 4 fate that may be amplified with prolonged MDSC exposure. This coordinated metabolic reprogramming and selective expansion of inflammation-enriched clusters reveals how MDSC exposure reshapes stromal composition of the PDAC TME.

## 4. Discussion

A key feature of our concave-bottom microfluidic platform is its ability to preserve 3D architecture, which mirrors *in-vivo* TME, while maintaining a stable laminar flow that allows our immune cells to infiltrate the tumor in gradients. Unlike conventional 2D *in-vitro* models, where immune cells are simply added on top of a monolayer and uniformly interact with the entire sample, our platform allows MDSCs to circulate through the channels, exposing CAFs dynamically and selectively, which is critical for observing targeted MDSC signaling and specific precursor CAF activation. These carefully engineered spatial and perfusion dynamics of our platform revealed previously hidden aspects of stromal plasticity and immune–stromal targeted signaling. While dynamic immuno–stromal interactions occur naturally in murine PDAC models, their complexity and heterogeneity also make it difficult to isolate precursor CAF populations or to dissect the events specifically driven by MDSC infiltration. Similarly, existing MPS often lack the combination of perfusable three-dimensional architecture, and reversibly-sealable channels that allow for both real-time observations, and extraction of the embedded samples for terminal analysis, like single cell RNA sequencing. These engineered characteristics validate our platform as a unique and reliable model for recapitulating key aspects of the *in vivo* TME that other PDAC models fail to mimic, while maintaining a level of resolution and control over the cellular environment unattainable with standard *in-vitro* or *in-vivo* approaches. Furthermore, the transparent acrylic architecture of our platform enables real-time live imaging of immune–stromal interactions, allowing individual MDSCs to be tracked as they migrate toward the CAF-tumor spheroid over time, something that static or endpoint-only platforms cannot provide (supplementary figure 4). This will enable systematic mapping of MDSC migration dynamics and allow us to directly visualize how disrupting specific signaling axes alters immune cell recruitment and behavior within the TME.

The stromal–immune dynamics of PDAC have been studied utilizing multiple types of model, each with key limitations. Foundational Matrigel-based co-culture studies by Öhlund *et al* established that spatially distinct iCAF and myCAF populations arise from tumor-derived signals, while patient-derived organoid-CAF systems have since extended this to interrogate chemoresistance and stroma-driven transcriptional reprogramming [14, 66]. *In vivo*, KPC genetically engineered mouse models have established MDSCs as a dominant immunosuppressive leukocyte population in PDAC, but the complexity of these systems makes it difficult to isolate the specific contribution of individual immune populations to stromal reprogramming [67]. At the platform level, Tao *et al* recently developed a state-of-the-art vascularized PDAC-on-chip model that recapitulates tumor–stroma interactions under perfusion conditions, representing a significant advance in physiological relevance for microfluidic PDAC modeling [68]. Building on this and related microfluidic approaches, microfluidic PDAC-on-chip models have demonstrated that the stroma forms a physical and biochemical barrier to immune infiltration and can recapitulate macrophage polarization under flow conditions, while bioengineered tumor–stroma platforms have reproduced hallmark immunosuppressive features including T cell exhaustion [69–71]. Notably, however, even the most advanced platforms including that of Tao *et al* have not specifically isolated the contribution of individual immune populations, such as MDSCs, to CAF polarization and stromal fate commitment. Single-cell transcriptomic profiling of human PDAC has further revealed at least nine CAF subtypes with distinct functional and prognostic significance, underscoring the depth of stromal heterogeneity that remains poorly captured by existing models [68]. While these platforms advance our understanding of stromal–immune dynamics, they have yet to investigate the isolated immune signals and mechanisms that drive CAF polarization. Our platform is uniquely positioned to close this gap by combining perfusable 3D architecture with the spatial and temporal resolution needed to isolate targeted MDSC-reprogramming of the stroma from the broader immune landscape of the TME.

We demonstrate that MDSCs actively induce the transition of CAFs toward an iCAF polarization as well as demonstrate that this effect was not uniform across the fibroblast population. MDSCs appeared to selectively promote inflammation in specific CAF subsets, suggesting a level of target specificity within the stromal cells. Further work is needed to identify the signaling pathways and mechanisms responsible for MDSC-driven inflammation. Specifically, we will utilize MDSCs and CAFs with key knockout signaling mediators, such as *Il1β, Il6*, and *Stat3* components, to determine which pathways are necessary for iCAF polarization. Complementary studies using IL-6 and IL-1*β* inhibitor drugs will further validate these findings and help delineate paracrine versus contact-dependent effects of MDSCs. Together, these mechanistic studies will help us understand how MDSCs reprogram CAFs and uncover therapeutic targets to block or reverse CAF inflammation in PDAC.

The transcriptional and trajectory analyses presented in section 3.4 reveal a previously undescribed mechanism by which MDSCs reshape the PDAC microenvironment. MDSCs appear to selectively amplify a fate-primed progenitor population and accelerate its differentiation into an inflammatory-enriched state, rather than broadly activating all stromal cells. These findings align with the emerging evidence that CAF subpopulations exist along a transcriptional range rather than as fixed identities, and that the balance between these states can be actively modulated by immune inputs from the TME [72–74]. The dramatic expansion of cluster 4 under MDSC treatment, alongside significant suppression of MYC targets and trending enrichment of inflammatory response pathways, is consistent with a shift away from proliferative programs toward a secretory inflammatory CAF state resembling the iCAF phenotype described in PDAC by Öhlund *et al* [14]. Future work incorporating cytokine profiling and immune cell co-culture experiments are needed to establish whether the MDSC-driven expansion of cluster 4 contributes to the immunosuppressive and pro-tumerogenic environment of PDAC. The co-activation of mTOR and hypoxia signaling across multiple CAF clusters suggests that MDSC exposure drives a coordinated reprogramming of the stromal compartment. Given that mTOR activity in PDAC CAFs has been shown to drive their pro-tumorigenic secretory function including IL-6 production, and that hypoxia promotes iCAF induction in PDAC through HIF-1*α*-dependent mechanisms, the metabolic reprogramming observed here may reflect an MDSC-driven conditioning of the CAF compartment toward a more inflammatory and secretory state [46, 75, 76]. The modest but consistent MDSC-driven increase in fate probability toward cluster 4 across all remaining CAF clusters further suggests that this is not an effect from a single subpopulation, but rather a compartment-wide bias towards inflammation that MDSCs exploit. Collectively, these findings position MDSCs not merely as immunosuppressive effectors acting on T cells, but as active architects of stromal identity in PDAC. Further work using lineage tracing and metabolic perturbations is needed to confirm the trajectory and role of progenitor CAF populations in iCAF polarization and define potential targets for intervention.

Our current 48 h timeframe may also be obscuring slower signaling events or delayed phenotypic transitions, limiting our ability to fully capture the switch of progenitor CAFs into an inflammatory state. Future work extending the duration of incubation and sampling across multiple time points will therefore be critical for uncovering the complete dynamics of CAF–MDSC interactions and their downstream impact on tumor progression. Additionally, we acknowledge that ECM is a critical component of the *in vivo* PDAC TME that may amplify or modulate MDSC-driven CAF polarization. In future iterations of the platform incorporating physiologically relevant ECM components will be essential for validating these findings in a more complete microenvironmental context. However, the absence of extracellular matrix in our platform was a deliberate design choice to isolate direct immune–stromal crosstalk without the confounding influence of matrix-mediated cytokine sequestration, limited diffusion gradients, and possible mechanical cues. This isolation will enable precise identification of the key signaling pathways mediating MDSC–CAF communication that could serve as therapeutic targets to attenuate tumor progression. Furthermore, our self-assembled spheroids emerge from co-seeding tumor cells and CAFs, instead of engineered organoids where CAFs may be seeded into an outer shell. We observed some proof of CAF localization to the periphery of self-assembled spheroids (supplementary figure 5). These preliminary observations do not robustly demonstrate the classic PDAC architecture where CAFs surround a tumor core. Lack of a stromal barrier may additionally influence immune cell infiltration dynamics. Future studies incorporating layered spheroids will be essential for determining how stromal positioning shapes the immune-driven reprogramming we observe.

Several limitations must also be considered when interpreting the single-cell RNA sequencing analysis. First, each condition was represented by a single pooled sequencing library derived from 15 spheroids, which limits the statistical robustness of differential expression findings. This pooling strategy was necessitated by the cell number requirements of the 10× Genomics platform and is consistent with approaches used in organoid-based scRNA-seq studies [77]; however, future experiments incorporating multiple independent sequencing libraries per condition will be essential for confirming the reproducibility and generalizability of the transcriptional states and CAF polarization dynamics we identify. Second, the trajectory and fate analyses, while internally consistent across multiple orthogonal methods, are inferential in nature. RNA velocity, CellRank, and PAGA model transcriptional dynamics from static single-cell snapshots and cannot directly observe cell state transitions in real time. Further work using lineage tracing and time-course scRNA-seq experiments is needed to confirm the progenitor-to-iCAF relationship proposed. Additionally, the GSEA pathway enrichment analysis, including the inflammatory response pathway in cluster 4 (padj = 0.076) and mTORC1 signaling in cluster 2 (padj = 0.092), reached only trending significance, and conclusions drawn from these findings should be interpreted with appropriate caution pending replication in larger or independent datasets. Lastly, while the convergence of velocity confidence, fate probability, and PAGA transition data consistently supports a cluster 6-to-cluster 4 differentiation axis, the absence of significant pathway enrichment in cluster 6 itself limits our ability to define its identity through transcriptional programs alone, and the mechanisms governing its progenitor-like behavior remain to be elucidated. Addressing these limitations will not only validate precursor-to-iCAF transitions but also expand our platforms capabilities to investigate the full extent of the immuno–stromal interactions in the TME, their downstream effects on tumor biology, and highlight potential therapeutic interventions.

Beyond modeling PDAC, our microfluidic platform has broad potential for studying other immune–stromal interactions, cancer types, fibrotic disease, and inflammatory conditions. Our platform versatility and perfusable 3D architecture could be applied to other solid tumors such as breast or lung cancer, where fibroblast-immune crosstalk drives tumor progression, or to fibrotic organs like the liver, lung, and heart, where stromal activation contributes to disease pathology. Additionally, the platform can be used to model autoimmune environments, like type I diabetes, by co-culturing pancreatic islets with activated immune cells, to enable precise and real-time investigation of beta cell metabolic stress, cytokine-mediated dysfunction, and paracrine interactions that influence insulin secretion and glucose homeostasis. Similarly, our platform could be leveraged to study liver toxicity by incorporating primary hepatocytes along with Kupffer cells and hepatic stellate cells, allowing dynamic assessment of drug-induced metabolic perturbations, oxidative stress responses, and paracrine signaling within a physiologically relevant 3D microenvironment. By allowing precise manipulation of the cellular microenvironment, signaling gradients, and temporal dynamics, the platform provides a generalizable tool for investigating complex *in-vivo* interactions and testing potential therapeutic interventions across multiple diseases and conditions.

In conclusion, we engineered a microfluidic platform that faithfully recapitulates key stromal–immune interactions in PDAC, revealing that MDSCs drive an inflammatory stromal landscape by selectively expanding a fate-committed progenitor population and accelerating its differentiation toward an inflammation-enriched CAF state. This process is accompanied by a coordinated reprogramming of the broader CAF compartment, including mTORC1 and hypoxia pathway co-activation, suggesting that MDSCs condition the broader stromal compartment for sustained pro-tumorigenic support. By combining microenvironmental control, real-time observation, and terminal analysis of incubated samples, the platform provides a powerful tool for dissecting the mechanisms underlying stromal plasticity and immune modulation in PDAC. Targeting the pathways that lead progenitor CAFs to achieve an inflammatory state could help attenuate tumor progression and thus make the TME more responsive to chemotherapy treatments. Likewise, targeting the pathways that mediate CAF inflammatory reprogramming could help attenuate stromal-driven immunosuppression, potentially improving the efficacy of immunotherapies. More broadly, these findings demonstrate that MDSCs can selectively reprogram stromal fate trajectories rather than simply activate existing populations, providing a framework for understanding TME adaptability, critical for designing the next generation of therapeutics targeting both the immune and stromal compartments of PDAC.

## Data Availability

All data that support the findings of this study are included within the article (and any supplementary files). Supporting information available at https://doi.org/10.1088/1758-5090/ae7b09/data1.
